# Chitosan-Loaded *Lagenaria siceraria* and *Thymus vulgaris* Potentiate Antibacterial, Antioxidant, and Immunomodulatory Activities against Extensive Drug-Resistant *Pseudomonas aeruginosa* and Vancomycin-Resistant *Staphylococcus aureus*: In Vitro and In Vivo Approaches

**DOI:** 10.3390/antiox13040428

**Published:** 2024-03-30

**Authors:** Selwan M. Taha, Norhan K. Abd El-Aziz, Adel Abdelkhalek, Ioan Pet, Mirela Ahmadi, Sameh M. El-Nabtity

**Affiliations:** 1 Department of Pharmacology, Faculty of Veterinary Medicine, Zagazig University, Zagazig 44511, Egypt or selwaan.m24@vet.zu.edu.eg (S.M.T.); samehelnabtity@gmail.com (S.M.E.-N.); 2 Department of Microbiology, Faculty of Veterinary Medicine, Zagazig University, Zagazig 44511, Egypt; 3 Food Safety, Hygiene and Technology Department, Faculty of Veterinary Medicine, Badr University in Cairo (BUC), Badr City 11829, Egypt; adel.abdelkhalek@buc.edu.eg; 4 Department of Biotechnology, Faculty of Bioengineering of Animals Resources, University of Life Sciences “King Mihai I” from Timisoara, 300645 Timisoara, Romania; mirelaahmadi@usvt.ro

**Keywords:** *Lagenaria siceraria*, *Thymus vulgaris*, chitosan nanoparticles, *P. aeruginosa*, VRSA

## Abstract

Antimicrobial resistance poses considerable issues for current clinical care, so the modified use of antimicrobial agents and public health initiatives, coupled with new antimicrobial approaches, may help to minimize the impact of multidrug-resistant (MDR) bacteria in the future. This study aimed to evaluate the antimicrobial, antioxidant, and immunomodulatory activities of *Lagenaria siceraria*, *Thymus vulgaris*, and their chitosan nanocomposites against extensive drug-resistant (XDR) *Pseudomonas aeruginosa* and vancomycin-resistant *Staphylococcus aureus* (VRSA) using both in vitro and in vivo assays. The in vitro antimicrobial susceptibilities of *P. aeruginosa* and VRSA strains revealed 100% sensitivity to imipenem (100%). All *P. aeruginosa* strains were resistant to cefoxitin, cefepime, trimethoprim + sulfamethoxazole, and fosfomycin. However, *S. aureus* strains showed a full resistance to cefoxitin, amoxicillin, ampicillin, erythromycin, chloramphenicol, and fosfomycin (100% each). Interestingly, all *S. aureus* strains were vancomycin-resistant (MIC = 32–512 μg/mL), and 90% of *P. aeruginosa* and *S. aureus* strains were XDR. The antimicrobial potential of *Lagenaria siceraria* and *Thymus vulgaris* nanocomposites with chitosan nanoparticles demonstrated marked inhibitory activities against XDR *P. aeruginosa* and VRSA strains with inhibition zones’ diameters up to 50 mm and MIC values ranging from 0.125 to 1 μg/mL and 1 to 8 μg/mL, respectively. The results of the in vivo approach in male Sprague Dawley rats revealed that infection with *P. aeruginosa* and *S. aureus* displayed significant changes in biochemical, hematological, and histopathological findings compared to the negative control group. These values returned to the normal range after treatment by chitosan nanoparticles, either loaded with *Lagenaria siceraria* or *Thymus vulgaris*. Real-time quantitative polymerase chain reaction (RT-qPCR) findings presented significant upregulation of the relative expression of the *IL10* gene and downregulation of the *IFNG* gene throughout the experimental period, especially after treatment with chitosan nanoparticles loaded either with *Lagenaria siceraria* or *Thymus vulgaris* in comparison to the positive control groups. In conclusion, this is the first report suggesting the use of *Lagenaria siceraria* and *Thymus vulgaris* nanocomposites with chitosan nanoparticles as a promising contender for combating XDR *P. aeruginosa* and VRSA infections as well as a manager for inflammatory situations and oxidative stress-related disorders.

## 1. Introduction

Antimicrobials serve as the primary basis for the treatment of microbial infections. However, the misuse and overuse of antimicrobials are the main drivers in the development of drug-resistant pathogens [1]. The World Health Organization (WHO) has asserted that antimicrobial resistance (AMR) is one of the top ten worldwide public health concerns to humanity. The efficacy of antimicrobials may vanish within the span of five years due to genetic mutations [2]. Antimicrobial resistance raises the expense of healthcare, lengthens the hospital stays, and increases mortality rates. Among bacterial strains, antimicrobial resistance is considered a serious situation [3,4,5,6]. *P. aeruginosa* is amongst the most imperative opportunistic pathogens in animals and humans. It is ubiquitous and possesses high intrinsic resistance to antimicrobials due to the low outer membrane permeability and the existence of diverse multiple drug efflux pumps [7,8]. On the other hand, *Staphylococcus aureus* (*S. aureus*), a Gram-positive commensal bacteria, is common in healthy people’s nares and on their skin. However, *S. aureus* has the ability to invade internal tissues or the bloodstream, causing a variety of serious infections. *S. aureus* is a leading human pathogen that causes a wide range of clinical symptoms ranging from relatively mild skin and soft tissue infections to serious and even fatal systemic illnesses [9]. It continues a challenging public health concern due to the rise and spread of multidrug-resistant strains such as methicillin-resistant *S. aureus* (MRSA) and vancomycin-resistant *S. aureus* (VRSA), which were first identified in the U.S. in 2002 [10]. 

Currently, it has become essential to screen affordable, accessible, safe, and effective therapeutics from a variety of medicinal plants, such as herbs, for their potential antimicrobial activities. Thyme (*Thymus vulgaris*) is one of the most valuable medicinal plants that exhibits bactericidal and bacteriostatic effects against many micro-organisms. The antimicrobial properties of thyme oil are mainly related to its high phenolic contents, such as carvacrol and thymol [11]. Furthermore, *Lagenaria siceraria* belongs to the *Cucurbitaceae* family and is used as an edible medicinal plant because the fruit contains vitamins C and B complex, proteins, and fatty acids. *Lagenaria siceraria* essential oil is found to be very important in the world market due to its medicinal properties [12]. This plant has been used for many pharmacological purposes due to its antihepatotoxic, analgesic, anti-inflammatory, hypolipidemic, immunosuppressive, immunomodulatory, cytotoxic, diuretic, cardioprotective, anti-hyperlipidemic, anti-obesity, and antioxidant properties [13]. Deacetylated chitin, or chitosan, is a naturally occurring substance that can be found in fungus cell walls and crustacean shells [14]. The broad-spectrum antimicrobial activity of chitosan may be attributed to (1) attachment to the negatively charged bacterial cell wall, disrupting the cell and changing the permeability of the cell membrane; (2) adhesion to DNA, which prevents DNA replication and ultimately leads to cell death; and (3) attachment to trace metal elements in an elective way, producing toxins and preventing microbial development chelating. Chitosan nanoparticles demonstrated low toxicity both in vitro and in vivo models. It has diverse applications in drug delivery for the treatment of neoplastic lesions, pulmonary and gastrointestinal diseases, and ocular infections [15,16].

Herein, in vitro and in vivo evaluation of the antimicrobial, antioxidant, and immunomodulatory potentials of *Lagenaria siceraria*, *Thymus vulgaris* plant extracts and their combination with chitosan nanoparticles were performed that may serve as basic structures for the development of novel therapy against the extensive drug-resistant (XDR) *P. aeruginosa* and VRSA.

## 2. Materials and Methods

### 2.1. Bacterial Strains

Two bacterial species, XDR *P. aeruginosa* and VRSA (n = 10 each), were gratefully acquired from the Department of Microbiology, Faculty of Veterinary Medicine, Zagazig University. The bacterial isolates were obtained from infrequent cases of bovine clinical mastitis at Zagazig City, Egypt. The presumptive bacterial isolates were confirmed by implementing the standard microbiological techniques [17]. *P. aeruginosa* isolates were cultivated on pseudomonas cetrimide agar (Oxoid, Cambridge, UK). Meanwhile, Baird–Parker agar supplemented with tellurite–egg yolk emulsion (Oxoid, UK) was used to isolate *S. aureus*. Genomic DNA was isolated from fresh bacterial isolates using the QIAamp DNA Mini kit (Qiagen, Hilden, Germany) following the manufacturer’s guidelines. Species-specific primer pairs (Metabion, Planegg, Germany) (Supplementary Table S1) [18,19] were used for the identification of the *oprL* gene of *P. aeruginosa*, whereas the *nuc* gene was amplified for the confirmation of *S. aureus*. 

### 2.2. Antimicrobial Susceptibility Testing of Bacterial Strains

The disc diffusion assay [20] was applied to test the susceptibilities of *P. aeruginosa* and *S. aureus* to a list of antimicrobials approved for both human and livestock use [21]. *P. aeruginosa* strains were tested against 17 antimicrobial discs of the following nine classes: aminoglycosides [gentamicin (CN, 10 µg), tobramycin (TOB, 10 µg), amikacin (AK, 30 µg), and netilmicin (NET, 30 µg)], carbapenems [imipenem (IMP, 10 µg) and meropenem (MRP, 10 µg)], cephalosporines [ceftazidime (CAZ, 30 µg) and cefepime (FEB, 30 µg)], quinolones [ciprofloxacin (CIP, 5 µg), levofloxacin (LEV, 5 µg), and nalidixic acid (NA, 30 µg)], β-lactamase inhibitors [piperacillin-tazobactam (PTZ, 100/10 µg)], sulphonamides [sulfamethoxazole + trimethoprim (SXT, 23.75/1.25 µg)], monobactams [aztreonam (ATM, 10 µg)], phosphonic acids [fosfomycin (FF, 50)], and polymyxins [polymyxin B (PB, 300 µg)].

*Staphylococcus aureus* strains were tested against 16 antimicrobial discs of the following 13 classes: aminoglycosides [gentamicin (CN, 10 µg) and streptomycin (S, 100 µg)], ansamycins [rifampin (RA, 30 µg)], penicillin [ampicillin (AMP 10 µg) and amoxicillin (AX, 30 µg)], glycopeptides [vancomycin (VA, 30 µg)], fluoroquinolones [ciprofloxacin (CIP, 5 µg)], tetracyclines [tetracycline (TC, 30 µg) and doxycycline (DO, 30 µg)], cephalosporins [cefoxitin (FOX, 30 µg)], sulphonamides [sulfamethoxazole + trimethoprim (SXT, 23.75/1.25 µg)], carbapenems [imipenem (IMP, 10 µg)], macrolides [erythromycin (E, 30 µg)], phenicol [chloramphenicol (C, 30 µg)], phosphoric acid [fosfomycin (FF, 50 µg)], and fusidane [fusidic acid (FA, 10 µg)]. The minimum inhibitory concentration (MIC) for vancomycin (Sigma-Aldrich, St. Louis, USA) was detected by the broth microdilution assay [22]. The antimicrobial susceptibility results were interpreted following Clinical and Laboratory Standards Institute (CLSI) criteria [23]. Calculation of the multiple antibiotic resistance (MAR) index for each strain was as follows: Number of antimicrobials to which the strain displayed resistance/Number of antimicrobials the strain has been exposed to, while the antimicrobial MAR index = Total number of resistance scored/(Total number of bacterial strains × Total number of antimicrobials tested). The American Type Culture Collection (ATCC)’s *P. aeruginosa* (ATCC 27,853) and *S. aureus* (ATCC 25,923) were used as quality controls. 

### 2.3. Natural Plants and Chitosan

Birdhouse gourd seeds (*Lagenaria siceraria*) and garden thyme leaves (*Thymus vulgaris*) were obtained from local markets and identified by a taxonomist at the Department of Botany, Faculty of Science, Zagazig University. In addition, low-molecular-weight chitosan (50–190 kDa) with 75–85% of deacetylation was obtained from Sigma-Aldrich, a St. Louis, Missouri-based company, USA.

### 2.4. Extraction of Lagenaria siceraria and Thymus vulgaris Essential Oils

For the isolation of *Lagenaria siceraria* essential oil [24], the seeds within the fruits were exposed by cutting them apart. These seeds were thoroughly collected, washed, allowed to dry in the shade for three days, and pulverized using the husk. The pulverized seeds (804.40 g) were steeped in 1.2 L of dichloromethane (Sigma-Aldrich, St. Louis, MO, USA) for 72 h at room temperature under frequent agitation to extract the seed oil. 

Isolation of *Thymus vulgaris* essential oil was carried out by the hydro distillation technique [25] using a modified Clevenger-type apparatus (Mayalab, Ambala Cantt, India) connected to a 4 L round-bottom and ground mouth flask. The extraction process was applied for 2 h while the solution was kept boiling. The hydrolat (water and oil) was collected and centrifuged at 321.8× *g* for 5 min in a Fanem-Baby I Mod 206 centrifuge (Sao Paulo, Brazil) in order to separate the organic phase from the aqueous one. After being separated using a Pasteur pipette, the essential oil was put in a glass bottle and refrigerated [26].

### 2.5. Gas Chromatography–Mass Spectrometry Analysis (GC-MS)

The GC-MS system (Agilent Technologies, Santa Clara, CA, USA) was equipped with a gas chromatograph (7890B) and mass spectrometer detector (5977A) at Central Laboratories Network, National Research Centre, Cairo, Egypt. The GC was equipped with a DB-5MS column (30 m × 0.25 mm internal diameter and 0.25 μm film thickness). Analyses were carried out using Helium as the carrier gas at a flow rate of 3.0 mL/min at a splitless injection volume of 1 µL and the following temperature program: 40 °C for 1 min; rising at 10 °C/min to 200 °C and held for 1 min; rising at 20 °C /min to 220 °C and held for 1 min; rising at 30 °C /min to 320 °C and held for 3 min. The injector and detector were held at 250 °C and 320 °C, respectively. Mass spectra were obtained by electron ionization (EI) at 70 eV using a spectral range of *m*/*z* 50–550 and solvent delay of 3 min. The mass temperature was 230 °C and Quad 150 °C. Identification of different constituents was determined by comparing the spectrum fragmentation pattern with those stored in Wiley and National Institute of Standards and Technology (NIST) Mass Spectral Library data.

### 2.6. Preparation of Essential Oil-Loaded Chitosan Nanoparticles [27]

Encapsulation of each essential oil into chitosan nanoparticles was completed in two steps; oil-in-water emulsification and ionic gelation protocol as presented previously [28]. Chitosan solution (1% *w*/*v*) was prepared via suspending the chitosan in 1% *v*/*v* acetic acid for 60 min of sonication (Kunshan Instrument Co., Ltd., Suzhou, Jiangsu, China). This solution was filtered through a 1 μm pore size filter to remove the undissolved chitosan.

An amount of 80 mg tween 80 was added to 50 mL chitosan solution, and the pH was adjusted to 4.2 by NaOH solution 2 N. The mixture was agitated at 50 °C for 90 min to produce a homogeneous solution. Different amounts of *Lagenaria siceraria* and *Thymus vulgaris* oils were added to this mixture to obtain 1:1. For achieving an oil-in-water emulsion, the oil phase was dropped continuously into the chitosan aqueous solution during homogenization (T25, IKA, Burladingen, Germany) in an ice-bath at 13,000 rpm for 10 min. Finally, sodium tripolyphosphate (TPP; Sigma-Aldrich, St. Louis, MO, USA) was supplied to influence the ionic gelation of chitosan. Agitation was continuously applied for 40 min. The particles were collected by centrifugation at 9000× *g* for 30 min at 4 °C and then washed with deionized water several times. These suspensions were dried immediately by a freeze dryer (Cryodos 50/230 V, Telstar, Madrid, Spain) at −32 °C for 42 h.

### 2.7. Characterization of Essential Oil-Loaded Chitosan Nanoparticles

Transmission electron microscopy (TEM) analysis was performed to inspect the surface morphological structure of chitosan nanoparticles loaded with either *Lagenaria siceraria* or *Thymus vulgaris* [29]. Furthermore, Fourier-transform infrared (FTIR) spectroscopy was applied to interrogate the functional groups of loaded and unloaded chitosan nanoparticles as previously described [30].

### 2.8. Antibacterial Activity of Chitosan Nanoparticles Loaded Either Lagenaria siceraria or Thymus vulgaris

The agar well diffusion test was carried out as described elsewhere [31]. Bacterial suspensions were prepared from fresh cultures in sterile saline and adjusted to an optical density of 0.5 McFarland (1.5 × 10^8^ CFU/mL), and then 0.1 mL was transferred to Mueller Hinton agar plates (MHA; Oxoid, Hampshire, UK) and spread with cotton swabs. Wells were performed in the agar plate by using a cork borer (8 mm in diameter), and 100 µL of each tested agent at concentrations 100%, 60%, and 20% (*w*/*v*) were pipetted into each well. Sterile distilled water was considered a negative control, while imipenem was used as a positive control for the bactericide action. The agar plates were incubated at 35 °C for 24 h, and the isolates with inhibition zone diameters ≥ 8 mm were considered susceptible. The experiments were carried out in triplicate. The broth microdilution assay was performed for the determination of minimum inhibitory concentration (MIC) and minimum bactericidal concentration (MBC) of each extract against bacterial strains under study [22]. Moreover, the MIC50 and MIC90 were calculated using an orderly array method [32].

### 2.9. Experimental Animals

Sixty-six Sprague Dawley male rats, 8 weeks old, with an average weight of 156.25 g, were provided from the Animal Laboratory House, Faculty of Veterinary Medicine, Zagazig University. All rats were housed in stainless steel cages in a pathogen-free environment maintained at a managed temperature (21–24 °C) with a 12 h light–dark cycle and a 50–60% relative humidity. Rats were acclimated two weeks prior to use. Throughout the experimental period, each cage contained feeders and pottery to supply free access to feed and fresh water, respectively. The diet formulation was considered to cover all the recommended nutrient requirements for rats, according to the National Research Council (NRC) [33]. Zagazig University Institutional Animal Care and Use Committee (ZU-IACUC) approved this study (approval number ZU-IACUC/2/F/138/2021).

### 2.10. Experimental Design

**Figure 1:**
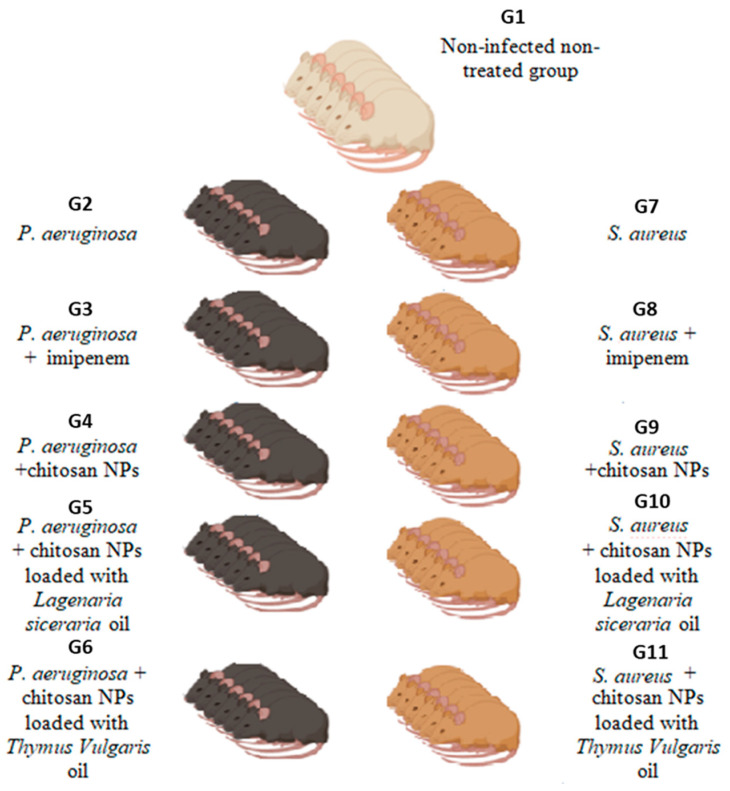
Experimental design for in vivo evaluation of *Thymus vulgaris*, *Lagenaria siceraria,* and their nanocomposite activities using sixty-six male Sprague Dawley rats. G, group; NP, nanoparticles.

Sixty-six male Sprague Dawley rats were randomly divided into eleven groups, six each (Figure 1). Group 1 was the negative control (non-infected, non-treated). Group 2 (the positive control) received *P. aeruginosa* (3 × 10^8^ colony forming units, CFU; intraperitoneal, IP) [34]. Group 3 received *P. aeruginosa* infectious dose and treated with imipenem (45 mg/kg body weight; intramuscular, IM). Group 4 received *P. aeruginosa* then treated with chitosan nanoparticles (5 mg/kg body weight; IP) [35]. Group 5 received *P. aeruginosa* and was treated with chitosan nanoparticles loaded with *Lagenaria siceraria* (5 mg/kg body weight; IP). Group 6 received *P. aeruginosa* and treated it with chitosan nanoparticles loaded with *Thymus vulgaris* oil (5 mg/kg body weight; IP). Group 7 was the positive control received *S. aureus* (6 × 10^8^ CFU, IP) [36]. Group 8 received the *S. aureus* infectious dose and treated with imipenem (45 mg/kg body weight; IM). Group 9 received *S. aureus* and was treated with chitosan nanoparticles (5 mg/kg body weight; IP). Group 10 received *S. aureus* and treated with chitosan nanoparticles loaded with *Lagenaria siceraria* (5 mg/kg body weight; IP), and group 11 received *S. aureus* and treated with chitosan nanoparticles loaded with *Thymus vulgaris* oil (5 mg/kg body weight; IP).

### 2.11. Biochemical and Serum Analysis

Two weeks post-challenge, serum samples were collected to evaluate the liver function markers such as aspartate aminotransferase (AST), alanine aminotransferase (ALT), and albumin as well as kidney function parameters such as blood urea and creatinine as described elsewhere [37]. Selective oxidative markers were assessed using colorimetric commercial kits purchased from Biodiagnostic Co. (Cairo, Egypt). Glutathione peroxidase (GPX), superoxide dismutase (SOD), and malondialdehyde (MDA) levels were determined following the methods described previously [38,39,40].

### 2.12. Measurement of Hematological Parameters

Hematological parameters, including hemoglobin (Hb) concentration, red blood cells (RBCs), and white blood cells (WBCs), were measured using the cell blood counter (Celltac α MEK6550, Nihon Kohden Company, Tokyo, Japan) as previously documented [37].

### 2.13. Enumeration of Bacteria

All samples were processed for isolation of *P. aeruginosa* and *S. aureus* as previously described [41]. Briefly, 1 g of each liver sample was mixed with 10 mL of buffered peptone water (Oxoid, Cambridge, UK) and then mixed for 2 min in a stomacher (Stomacher Lab-Blender 400; Seward, London, UK). Tenfold serial dilutions were prepared, and 0.1 mL of each diluted sample was inoculated onto the agar plates and incubated for 24 h at 37 °C. Baird-Parker agar (Oxoid, Cambridge, UK) provided with tellurite-egg yolk–emulsion (Oxoid, Cambridge, UK) was used for counting *S. aureus*. Whereas cetrimide agar base (Oxoid, Cambridge, UK) supplemented with nalidixic acid (15 mg/L) and cycloheximide (200 mg/L) was used for enumerating *P. aeruginosa*. The total viable counts were determined and computed as log_10_ CFU/g of the sample.

### 2.14. Pathological Examination

Liver specimens of represented rats were collected from all experimental groups two weeks from the beginning of the experiment, fixed in 10% neutral buffered formalin solution, dehydrated in gradual alcohol (70–100%), cleared in xylene, and embedded in paraffin. The paraffin sections (five-microns thickness) were prepared and stained with hematoxylin and eosin (HE) dyes and then examined microscopically [42].

### 2.15. Real-Time Quantitative Polymerase Chain Reaction (RT–qPCR)

Total RNA extraction was carried out utilizing the QIAamp RNeasy Mini kit (Qiagen, GmbH, Hilden, Germany). RT–qPCR was applied in triplicates to detect the expression patterns of *IL10* and *IFNG* genes in tissue specimens of rats after two weeks from the beginning of the experiment using a one-step RT-PCR kit with SYBR green (Qiagen, GmbH, Hilden, Germany) and oligonucleotide primer sequences listed in Supplementary Table S1. Reaction mixtures were incubated for 30 min at 50 °C, followed by a cycle of 94 °C for 15 min, 40 cycles of 94 °C for 15 s, 60 °C for 30 s, and 72 °C for 30 s (for *β. actin* and *IL10* genes) [43,44]; 94 °C for 15 s, 55 °C for 30 s and 72 °C for 30 s (for *IFNG* gene) [45]. Melting curves were created by a cycle of 94 °C for 1 min, 60 °C for 1 min (*B. actin* and *IL10* gene), 55 °C for one min (*IFNG* gene), and 94 °C for 1 min. The relative mRNA expression quantitation was normalized to the constitutive expression of the *β-actin* housekeeping gene. The comparative 2^−ΔΔct^ method was performed to compute the fold changes in the transcription levels of targeted genes in treated groups relative to their levels in the untreated ones [46].

### 2.16. Statistical Analysis

The data in this study were edited using Microsoft Excel (Microsoft Corporation, Redmond, WA, USA). To assess the normality, Levene and Shapiro–Wilk tests were conducted, following the methodology described elsewhere [47]. Significant differences in antimicrobial resistance of bacterial strains were tested via Fisher’s Exact Test. The Kruskal–Wallis test was used to analyze the significant differences in antimicrobial activities of examined extracts or nanoemulsions. Significant effects of treatments throughout the experimental groups were evaluated using a One-way ANOVA analysis (PROC ANOVA; SAS Institute Inc., Cary, NC, USA) [48], with a significance level (α) set at 0.05. If a significant effect was detected, pairwise comparisons between means were performed using Tukey’s test. Statistical significance between means was considered at a *p*-value below 0.05. Graphs were generated using GraphPad Prism software version 9.0 (GraphPad, La Jolla, CA, USA). The results were expressed as means ± standard error (SE).

## 3. Results

### 3.1. Antibiogram of P. aeruginosa and VRSA Strains

The in vitro antimicrobial susceptibilities of *P. aeruginosa* strains (n = 10) against 17 commonly used antimicrobials of 9 classes are presented in Table 1. The results revealed high susceptibility of tested strains to imipenem (100%). However, all *P. aeruginosa* strains were resistant to cefoxitin, cefepime, trimethoprim + sulfamethoxazole, and fosfomycin. On the other hand, *S. aureus* strains were tested for their susceptibility to 16 antimicrobial agents of 13 classes. As presented in Table 1, *S. aureus* strains showed absolute susceptibility to imipenem (100%), whereas a full resistance (100%) was observed toward cefoxitin, amoxicillin, ampicillin, erythromycin, chloramphenicol, and fosfomycin. All *S. aureus* strains were vancomycin-resistant, exhibiting MIC values ranging from 32 to 512 μg/mL. Interestingly, 90% of each *P. aeruginosa* and *S. aureus* strains were XDR. Statistical analysis revealed non-significant variations (*p* > 0.05) in the antimicrobial susceptibilities of bacterial strains to all tested antimicrobials except for meropenem, quinolones, and piperacillin-tazobactam (*p* < 0.001) for *P. aeruginosa* and rifamycin, vancomycin, and fusidane (*p* < 0.001) for VRSA strains.

### 3.2. Characterization of Lagenaria siceraria and Thymus vulgaris Using GC-MS Analysis

Gas chromatography–mass spectrometry (GC-MS) analysis of *Lagenaria siceraria* and *Thymus vulgaris* are indicated in Table 2 and Table 3. GC-MS analysis revealed that 9,12-Octadecadienoic acid (Z,Z)-, methyl ester (83.5%), and benzene, 1-methyl-3-(1-methylethyl)- (26.67%) are the major chemical compounds in each extract.

### 3.3. Transmission Electron Microscopy and FTIR for Characterization of the Nanoemulsion

**Figure 2:**
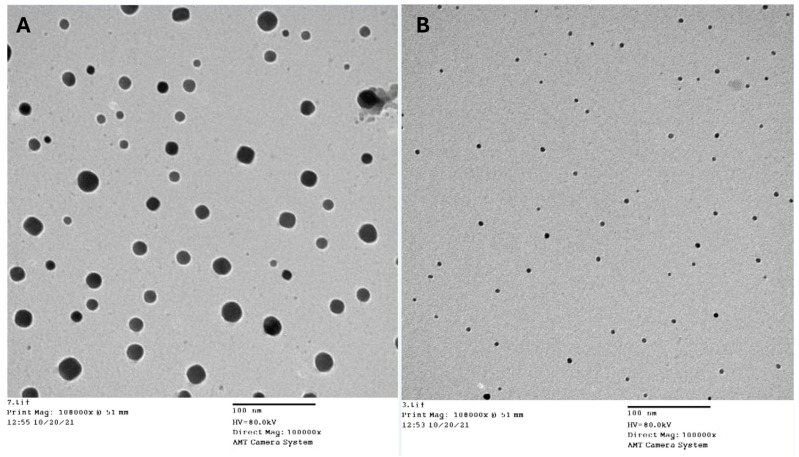
TEM micrographs of unloaded chitosan (**A**) and chitosan nanoparticles loaded with oil (**B**).

The TEM pictures of chitosan nanoparticles loaded with either *Lagenaria siceraria* or *Thymus vulgaris* oils are illustrated in Figure 2 at a reduced magnification scale of 100 nm. TEM morphological structure illustrated a large particle size, spherical-like structure, and particles in an agglomerated condition with non-aggregation. However, TEM showed that the nanoparticles were consistent in shape with a size range of ~54 nm of unloaded chitosan nanoparticles (Figure 2A) and ~250 nm in diameter (Figure 2B).

**Figure 3:**
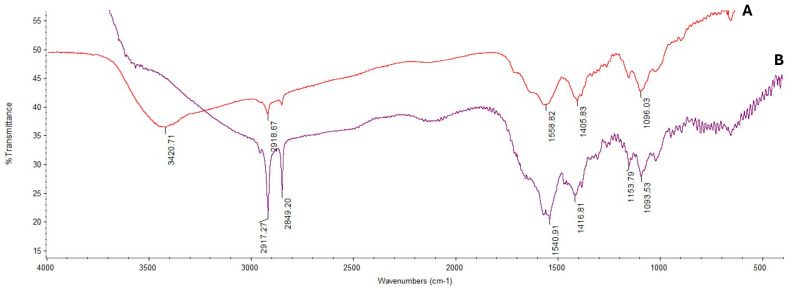
FTIR spectra of chitosan nanoparticles (**A**) and chitosan nanoparticles loaded with oil (**B**).

Fourier-transform infrared (FTIR) spectroscopy was used to examine the functional groups of either unloaded or loaded chitosan nanoparticles, as depicted in Figure 3. The O-H group of stretching vibrations produced a peak at 3420 cm^−1^ for the chitosan primary functional group. The absorption peaks existence at 1559 cm^−1^ and 1405 cm^−1^ are attributed to protonating the amino (NH_2_) group N-H bending vibration and the alkyl group C-H bending vibration. The absorption peaks at 1096 cm^−1^ and 655 cm^−1^ are attributed to the glucopyranose ring in the chitosan matrix due to the anti-symmetric stretching vibration of C-O-C bridges (Figure 3A). Furthermore, a peak shift and flattening were observed for chitosan nanoparticles loaded with *Thymus vulgaris* oil at 3370 cm^−1^, which is attributed to the O-H group stretching and may have resulted from the interactions between the molecules. A peak was observed at 2919 cm^−1^ belonging to the vibration of C-H in the benzene ring, the absorption peaks at 1630 cm^−1^, 1460 cm^−1^, and 1278 cm^−1^ belong to unequal stretching vibrations of the phenolic ring, and the peaks at 1247 cm^−1^, and 1098 cm^−1^ are related to the O-H and C-O vibrations of thymol (Figure 3B).

### 3.4. Antimicrobial Activities of the Essential Oils and Their Nanoemulsions against XDR P. aeruginosa and VRSA Strains

The antimicrobial potentials of *Lagenaria siceraria*, *Thymus vulgaris*, and their nanocomposites with chitosan nanoparticles against XDR *P. aeruginosa* and VRSA strains were evaluated by the agar well diffusion and broth microdilution techniques (Table 4). The essential oil diluent, tween 80, was inactive against all investigated strains. The results showed that *Lagenaria siceraria* and *Thymus vulgaris* exhibited minimum activities against *P. aeruginosa* and VRSA strains with inhibition zones’ diameters ranging from 2 to 20 mm and MICs of ≥32 μg/mL. However, marked inhibitory effects with inhibition zones’ diameters up to 50 mm were observed when chitosan nanoparticles loaded with *Lagenaria siceraria* or *Thymus vulgaris* with low MIC values ranging from 0.125 to 1 μg/mL and 1 to 8 μg/mL, respectively. For all screened strains, all tested antibacterial agents showed 2-fold higher MBC values than their recorded MICs, indicating their bactericidal activity. Moreover, the MIC50 and MIC90 values of *Lagenaria siceraria* (128 μg/mL both) and *Thymus vulgaris* (64 μg/mL and 128 μg/mL, respectively) were recorded, whereas those for chitosan nanoparticles loaded with *Lagenaria siceraria* (0.5 μg/mL and 1 μg/mL, respectively) or *Thymus vulgaris* (4 μg/mL and 8 μg/mL, respectively) against *P. aeruginosa* or VRSA strains were significantly decreased.

### 3.5. Results of the In Vivo Study

#### 3.5.1. Clinical Observation and Postmortem Lesions

The clinical symptoms were observed on the tenth day after bacterial challenge in the form of lack of appetite, depression, abdominal pain, breathing, wasting, diarrhea, increased respiration, lethargy, lying on sides, loss of righting reflex, porphyrin staining, tremors, anemia, and vent staining. Neither rats of groups 5 and 6 (infected by *P. aeruginosa* then treated with chitosan nanoparticles loaded with either *Lagenaria siceraria* or *Thymus vulgaris*) nor those of groups 10 and 11 (received *S. aureus* then treated with chitosan nanoparticles loaded with either *Lagenaria siceraria* or *Thymus vulgaris*) exhibited any clinical signs. Postmortem changes in the positive control groups showed abscess formation on the liver, kidney, spleen, and intestine, hepatitis, and hemorrhagic foci on the liver compared with rats treated by chitosan nanoparticles loaded with either *Lagenaria siceraria* oil or *Thymus vulgaris*, which showed normal postmortem examination (Supplementary Figure S1).

#### 3.5.2. Results of Biochemical and Hematological Parameters

As shown in Table 5, the positive control groups exhibited a significant (*p* < 0.05) increase in AST and ALT activities, serum urea, creatinine, albumin, and MDA levels together with decreasing SOD and GPX levels in comparison with respective parameters of the negative control group. Moreover, the positive control groups presented a dramatic change in the hematological parameters, showing a significant (*p* < 0.05) decrease in RBCs count and Hb, and an increase in WBCs, compared with the negative control. Certainly, therapeutic administration of the chitosan nanoparticles loaded with each of the essential oils under study succeeded in significantly enhancing the conditions of all analyzed parameters (Table 5 and Table 6).

#### 3.5.3. Microbial Count Analysis

On the 15th day of challenge, the total bacterial counts from liver tissues of rats of the positive control groups infected with either *P. aeruginosa* or *S. aureus* increased to 8 × 10^9^ CFU/mL and 1 × 10^10^ CFU/mL, respectively. Regarding rats that received *P. aeruginosa* and then treated with imipenem, the bacterial count increased to 3 × 10^9^ CFU/mL, whereas rats that received *S. aureus* were then treated with imipenem, the bacterial count increased to 8 × 10^8^ CFU/mL. Concerning rats treated with chitosan nanoparticles, the bacterial count increased to 4 × 10^8^ CFU/mL and 7.5 × 10^8^ CFU/mL, respectively.

**Figure 4:**
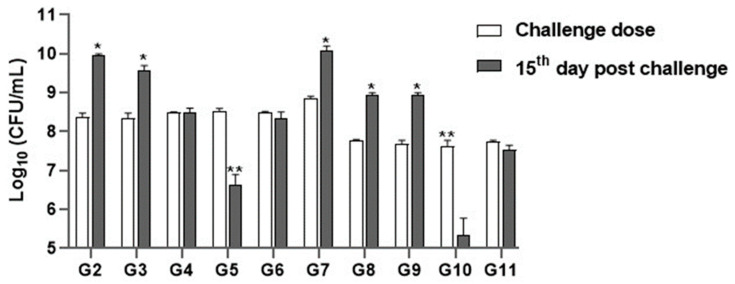
Recovery of bacteria from liver tissues by plate count. The data were expressed as log^10^ CFU/mL. Data were analyzed by a One-way ANOVA analysis and Tukey’s test. * indicates significant variation at *p* value < 0.05. ** indicates a highly significant difference at *p* value < 0.001.

On the other hand, the bacterial count from rats’ tissues that administrated *P. aeruginosa* and *S. aureus* then treated with chitosan nanoparticles loaded with *Lagenaria siceraria* oil decreased to 25 × 10^5^ and 6.0 × 10^5^ CFU/mL, respectively, whereas the bacterial count from those treated with chitosan nanoparticles loaded with *Thymus vulgaris* in combination with IP inoculation of *P. aeruginosa* or *S. aureus* revealed significant decrease to 15.0 × 10^7^ and 45 × 10^6^ CFU/mL, respectively (Figure 4).

#### 3.5.4. Histopathological Findings

**Figure 5:**
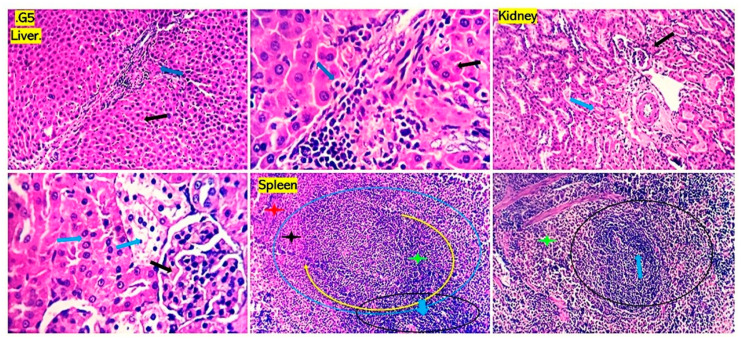
Photomicrograph from the liver, kidney, and spleen of rats received *Pseudomonas aeruginosa* and treated with chitosan loaded with *Lagenaria siceraria* oil, showing mild portal round cells (lymphocytes and plasma cells) aggregation (blue arrows). Scattered hepatocellular degenerative and apoptotic changes are seen (black arrows). A few renal tubules show focal tubular epithelial degeneration with intratubular hyaline cast formation (blue arrows) spleen demonstrating normal features of the white (black, yellow and blue circles, green and black stars) and red pulp (red star). H&E; 100×, 200×, and 400× power.

**Figure 6:**
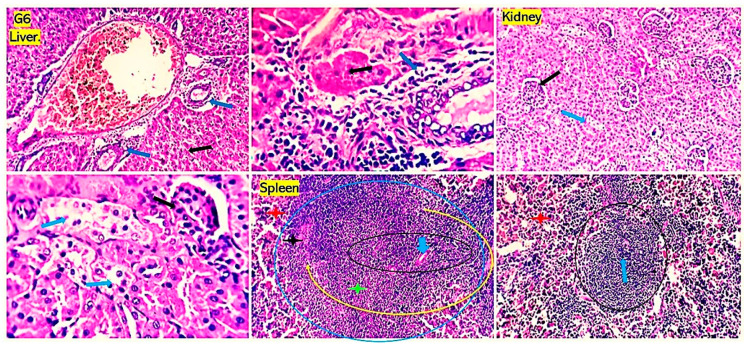
Photomicrographs from the liver, kidney, and spleen of rats received *Pseudomonas aeruginosa* and treated with chitosan loaded with *Thymus vulgaris* oil, showing mild portal vascular dilatation, blood engorgement, minimal biliary proliferation, and round cells (lymphocytes and plasma cells) aggregation (blue arrows). Scattered hepatocellular degenerative and apoptotic changes are seen (black arrows). The kidney shows a few tubules with focal tubular epithelial degenerative and early necrotic changes (blue arrows). Spleen reveals normal features of white (black, yellow and blue circles, blue arrow, green and black stars) and red pulp (red stars). H&E; 100×, 200×, and 400× power.

**Figure 7:**
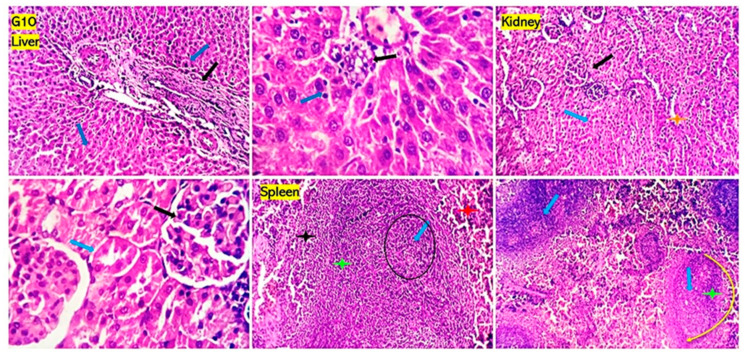
Photomicrographs from the liver, kidney, and spleen of rats received *Staphylococcus aureus* and treated with chitosan loaded with *Lagenaria siceraria* oil showing minimal biliary reaction (black arrow). Renal tubular epithelial regeneration is seen (orange star). A mild decrease in the population of the lymphoid cells of the white pulp (black and yellow circles. blue arrow and green stars), particularly the marginal zones (black star), and mild sinusoidal dilatation (red star) are seen in the spleen. H&E; 100x, 200x, and 400x power.

**Figure 8:**
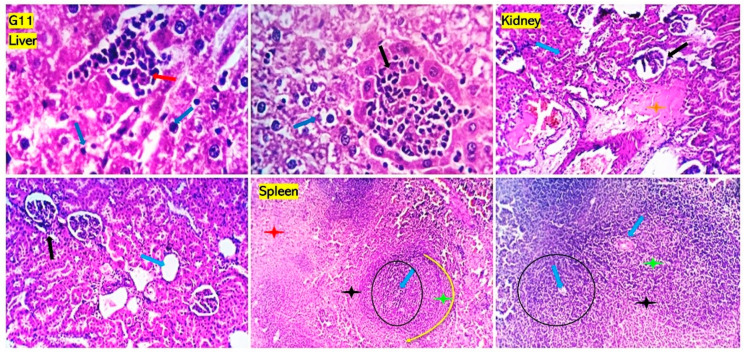
Photomicrographs from the liver, kidney, and spleen of rats received *Staphylococcus aureus* and treated with chitosan loaded with *Thymus vulgaris* oil showing extramedullary hematopoiesis (red and black arrows) and mild focal hepatocellular hydropic degeneration (blue arrows) in the liver. Focal glomerular shrinkage and lobulation (black arrows) beside perivascular and interstitial edema (orange star) are seen in the kidneys. A few tubules appear dilated (blue arrow). Spleen shows apparently normal histomorphology of both white (black, yellow circles. blue arrow, green and black stars) and red pulp (red star). A mild decrease population of the lymphoid cells of the white pulp marginal zones is seen (black star). H&E; 100×, 200×, and 400× power.

Examined sections from livers of the positive control groups for *P. aeruginosa* (Figure 5) and *S. aureus* (Figure 6) revealed dilated portal blood vessels, multifocal hemorrhages, and focal hepatocellular degenerative and necrotic changes beside portal inflammatory cellular infiltration with predominant lympho-plasmocytic types. Multifocal parenchymal necrotic areas surrounded and replaced by round cells (lymphocytes and macrophages) are a peculiar finding. Mild biliary proliferation could be detected in some cases. The Von Kupffer cells were hypertrophic in some instances. Scattered individual hepatocellular apoptosis with observable nuclear pyknosis and deep eosinophilic shrink cytoplasm with occasional apoptotic body formation was recorded. Renal lesions were represented by moderate vascular dilation, perivascular inflammatory edema, glomerular lobulation, and shrinkage with occasional epithelial crescent formation. Eosinophilic albuminous fluid accumulation was also seen in the surrounding intra and extracellular cellular spaces. Tubular epithelial degenerative and necrotic changes were encountered. Occasional intratubular hyaline cast formation were seen. Mild pyelitis could be observed in some cases. Splenic tissues of these groups showed severe dilation of splenic sinusoids, multiple hemorrhages, and decreased numbers of reticuloendothelial cells. Significant hemosiderosis in red and white pulp areas was recorded. Decreased population of the lymphoid cells of the germinal centers, mantle, and marginal zone were seen. The numbers of circulating and habitat mature lymphocytes of the splenic cords were quality diminished. However, the group that received *P. aeruginosa* and treated with chitosan loaded with *Lagenaria siceraria* oil or chitosan loaded with *Thymus vulgaris* (Figure 7 and Figure 8) gave better results than imipenem and chitosan groups (Supplementary Figure S2). Sections from different organs of these groups, which received chitosan loaded with *Lagenaria siceraria* oil or chitosan loaded with *Thymus vulgaris,* revealed apparently normal histomorphology of the liver with preserved hepatic parenchyma including cord arrangement, portal triads, and sinusoidal structural morphology besides supporting stromal framework. A few sections showed mild portal round cells (lymphocytes and plasma cells) aggregation. Kidney sections revealed apparently normal histological structures of the nephron units, the collecting ducts, the renal pelvis, and the stroma. Some sections showed a few tubules with focal tubular epithelial degeneration and intratubular hyaline cast formation. Sections from the spleens of these groups declared a normal splenic histomorphologic structure comparable to that of the control-free group with features of the white and red pulp.

#### 3.5.5. Transcriptional Modulation of IL10 and IFNG Cytokine Genes in the Experimental Groups

**Figure 9:**
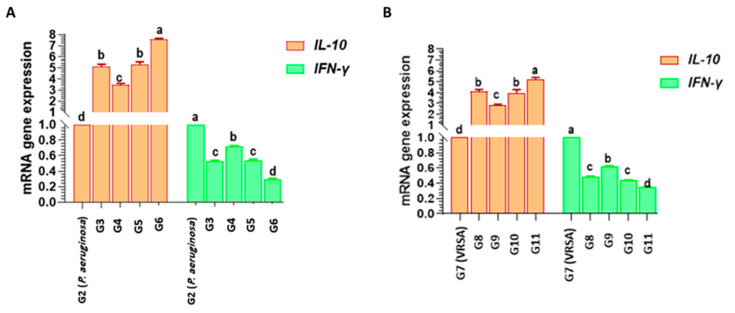
Changes in transcriptional modulation of *IL-10* and *IFN-γ* genes of rats infected by *P. aeruginosa* (**A**) and *VRSA* (**B**) strains throughout the experimental groups. The experimental design and nomenclature of each group are indicated in Figure 1. Different letters (a, b, c or d) above the columns indicate significant difference among the experimental groups (*p* value < 0.05).

The effects of chitosan nanoparticles loaded either with *Lagenaria siceraria* or *Thymus vulgaris* on transcriptional modulation of *IL10* and *IFNG* cytokine genes were assessed by RT-qPCR. Relative expressions (foldchange or fold-difference of expression levels) of the representative genes in the experimental groups were compared to those within the positive control groups, which were assigned a value of 1. The results of this study have a significant clinical impact. In particular, the *IL10* gene was found to be significantly upregulated throughout the experimental period, especially after treatment with chitosan nanoparticles loaded either with *Lagenaria siceraria* or *Thymus vulgaris*. However, the relative expression of the *IFNG* gene was significantly downregulated throughout the experimental groups in comparison with the positive control groups (Figure 9).

## 4. Discussion

It is acknowledged that the ability of either *P. aeruginosa* or *S. aureus* to be resistant is aggravated. These bacterial infections increase the morbidity and mortality rates in both humans and animals [49,50]. This growing concern has resulted in an insistent need for the development of alternative, simple, cost-effective, and ecologically friendly approaches to mitigate bacterial resistance [51]. Hence, this study focused on the evaluation of the antimicrobial, antioxidant, and immunomodulatory activities of *Lagenaria siceraria*, *Thymus vulgaris*, and their combination with chitosan nanoparticles against XDR VRSA and *P. aeruginosa* strains using both in vitro and in vivo assays. The overuse of antimicrobials has led to the rise of bacterial resistance; therefore, the first step in preventing the spread of antimicrobial resistance is to maintain reporting the resistance levels. Analyzing the antibiogram results of *S. aureus* and *P. aeruginosa* revealed that all tested strains were XDR, which was consistent with the results obtained previously [52,53].

Here, the agar well diffusion assay revealed that *Lagenaria siceraria* and *Thymus vulgaris* (100% each) showed lower activities against both bacterial species, with inhibition zone diameters ranging from 4 to 15 mm and 8 to 20 mm for *P. aeruginosa* and *S. aureus*, respectively. However, lower concentrations (60% and 20% each) exhibited low (2–10 mm) to no inhibitory activities against tested strains. These findings matched with the study of Ahmed and Ashiq [54] who stated that Lagenaria siceraria methanolic extract possessed a potency against *P. aeruginosa*, with an inhibition zone of 16.5 mm. The data of this study suggests that *Thymus vulgaris* and *Lagenaria siceraria* have potential applications as antibacterial agents against different species of pathogenic bacteria.

Herein, chitosan nanoparticles loaded with *Lagenaria siceraria* or *Thymus vulgaris* showed marked antibacterial activity against all *P. aeruginosa* and VRSA strains at all concentrations with inhibition zone diameters ranging from 35 to 50 mm (at 100% concentration). Moreover, the broth microdilution assay demonstrated general agreement with the disc diffusion results, which proved that chitosan nanoparticles loaded with *Lagenaria siceraria* or *Thymus vulgaris* are potent antibacterial agents against *P. aeruginosa* and VRSA strains with MIC values of 0.125–2 μg/mL and 1–8 μg/mL, respectively. Regarding the results obtained by Nagaraja and coauthors [55], *L. siceraria* had an antimicrobial activity against *S. aureus* with an MIC value of 0.25 × 10^3^ mg/mL, which differs from Diniz et al. [56], who found that thyme oil showed lower inhibitory activities against *P. aeruginosa* with MIC values ranging from 64 to 512 μg/mL. However, Keawchaoon and Yoksan [28] confirmed that chitosan nanoparticles with carvacrol exhibited antimicrobial activities against *S. aureus* compared to chitosan nanoparticles alone. Interestingly, our work reports the first study relating detailed antibacterial activities of *Lagenaria siceraria* or *Thymus vulgaris* chitosan nanocomposites against *P. aeruginosa* and VRSA strains, which have not yet been reported previously. However, a previous study was conducted on the combination of methanolic extract of *Lagenaria siceraria* with silver nanoparticles (AgNPs), which exhibited potent antimicrobial activities against all examined bacterial strains, including *P. aeruginosa* and *S. aureus* [57].

The results of the in vivo approach in male Sprague Dawley rats revealed that infection with *P. aeruginosa* and *S. aureus* displayed significant changes in biochemical, hematological, and histopathological findings compared to the negative control group. These values returned to the normal range after treatment by chitosan nanoparticles loaded with each of *Lagenaria siceraria* or *Thymus vulgaris*.

Liver enzymes such as ALT and AST are marker enzymes for liver function and integrity [58]. Hence, restoration of these enzymes to normal levels suggests that the liver is operating normally [59]. In the present study, serum biochemical analysis for liver functions revealed a significant increase in ALT and AST activities as well as a significant decrease in serum albumin levels in *P. aeruginosa* and *S. aureus*-infected rats compared to the negative control. The same results were achieved by Rahman et al. [60], who showed that the *S. aureus* challenge lowered the protein profile indices and elevated the liver enzyme activities. The *S. aureus* toxins may be responsible for destroying the hepatocytes. This is with the same line of the examined sections from the liver of the positive control groups for *P. aeruginosa* and *S. aureus* that revealed dilated portal blood vessels, multifocal hemorrhages, and focal hepatocellular degenerative and necrotic changes beside portal inflammatory cellular infiltration with predominant lymphoplasmacytic types. These results are consistent with Awad and coworkers [61], who demonstrated pathological alterations in the liver tissues of rats infected with MRSA.

On the other hand, rats treated with chitosan nanoparticles loaded with *Lagenaria siceraria* or *Thymus vulgaris* after inoculation with *P. aeruginosa* or *S. aureus* showed a significant decrease in serum ALT and AST activities. This is consistent with Amin and El-Kabany [62] who reported that serum biochemical parameters showed a significant decrease in AST and ALT activities as well as raised albumin and globulin levels of untreated infected rats compared to the negative control. Administration of thyme pre- or post-infection ameliorates the perceived changes, indicating the safe treatment of thyme on the organs of rats. The liver histopathology sections from different organs of rats that received chitosan nanoparticles loaded with *Lagenaria siceraria* or *Thymus vulgaris* revealed apparently normal histomorphology of the liver with preserved hepatic parenchyma including cord arrangement, portal triads, and sinusoidal structural morphology beside supporting stromal framework.

Herein, the results exposed a significant increase in the serum creatinine and urea levels in *P. aeruginosa* and *S. aureus* compared with negative control. The raised levels of blood urea nitrogen and serum creatinine are significant indicators of renal dysfunction, reflecting a drop in the glomerular filtration rate [63]. Therefore, our results indicated kidney dysfunction as the renal markers, particularly serum creatinine, is mostly a diagnostic tool for kidney function. The same results were achieved by Rahman et al. [60], who showed that *S. aureus* challenge increased the kidney function markers (creatinine and urea). In addition, Baraaj [64] stated that the blood collected from male rabbits injected by *P. aeruginosa* showed an elevation of plasma creatinine and urea levels compared with the control group.

Our biochemical results for the evaluation of kidney function were confirmed by the histopathological examination, where sections from the kidneys of *P. aeruginosa* and *S. aureus* infected rats showed renal lesions, which were represented by moderate vascular dilation, perivascular inflammatory edema, glomerular lobulation, and shrinkage with occasional epithelial crescent formation. Our findings ascertained those obtained by Baraaj [61], who reported several types of kidney damage in rabbits injected with *P. aeruginosa* DNA, such as infiltrated inflammatory cells, macrophages, and neutrophils around blood vessels and massive necrosis in the tubules. Similarly, Cheng et al. [65] recorded that kidney lesions were primarily marked by an influx of polymorphonuclear leukocytes (PMNs) and harbored no discernable organization of *Staphylococci*, most of which appeared to reside within PMNs.

However, the level of sera creatinine was significantly lower in rats treated with *Lagenaria siceraria*-loaded chitosan nanoparticles than all other treated groups (*p* < 0.05) with non-significant differences with the control group. These drops in the renal diagnostic parameters may be a result of improved renal function. Similar results were recorded by Rahmawati et al. [66] who found the ability of ethanolic extract of thyme to maintain the kidney function of diabetic read by measuring the serum urea and creatinine levels. The result of this study supports those previously obtained in another study [62] in which serum biochemical parameters exhibited a significant elevation in the activities of urea and creatinine of untreated infected rats compared to the negative control. Administration of thyme pre- or post-infection ameliorates the detectable changes, indicating the safe treatment of thyme on the organs of rats.

The histopathological findings in the kidney sections of rats treated with chitosan nanoparticles loaded with *Lagenaria siceraria* or *Thymus vulgaris* revealed apparently normal histological structures of the nephron units, the collecting ducts, renal pelvis, and stroma. Sections from the spleen of these groups declared a normal splenic histomorphologic structure comparable to that of the control-free group with features of the white and red pulp. The result of this study reinforced those previously obtained by Aref et al. [67], who stated that treatment with *Lagenaria siceraria* or melatonin after tramadol administration for a long term markedly changed the collagen content and other chemical components. Several studies have reported that phenolic and flavonoids present in Lagenaria siceraria have anti-inflammatory and antioxidant effects [68,69].

In this study, the administration of *P. aeruginosa* and *S. aureus* in rats showed significant changes in the oxidative stress markers. The results revealed a decrease in SOD and GPX and an increase in MDA levels compared to the negative control. Rostami et al. [70] stated that thyme oil decreased the NO and MDA levels, so thyme oil can improve the antioxidant capacity and decrease the oxidative stress of liver tissue. Notably, there is no published data regarding the pathological and antioxidant activities of *Lagenaria siceraria* or *Thymus vulgaris*-loaded chitosan nanoparticles to be discussed with the current findings.

Immune regulation plays an important role in protecting the host from the pathology related to bacterial infection. IL-10, which is produced by numerous innate and adaptive immune cells, including monocytes, macrophages, and T and B cells, is a prototypic immunoregulatory cytokine that is necessary for controlling/regulating the production of proinflammatory cytokines both in vitro and in vivo [71]. On the other side, IFNγ is a crucial modulator of immunity to intracellular microbes and is robustly induced upon bacterial infection. They can activate immune system cells, such as natural killer cells and macrophages, while also enhancing the speed of infection recognition by regulating antigen presentation to T lymphocytes [72,73]. The effects of chitosan nanoparticles loaded with *Lagenaria siceraria* oil or *Thymus vulgaris* on transcriptional modulation of *IL10* and *IFNG* cytokines genes was assessed by RT-qPCR. The *IL10* gene was found to be significantly upregulated throughout the experimental period, especially after treatment with chitosan nanoparticles loaded either with *Lagenaria siceraria* or *Thymus vulgaris*. However, the relative expression of the *IFNG* gene was significantly downregulated throughout the experimental groups in comparison with the positive control groups. The protective role of each plant extract may be ascribed to its higher content of flavonoids, which either scavenge free radicals or increase the production of GST. Our findings agreed with those obtained by the Wang team [74], who reported that managing microbe-induced immune suppressive factors, such as *IL-10* and *PD-L1*, may have profound clinical value, especially during immunizations, although the improvement in inflammatory responses might raise questions about the therapeutic value during infection. Van Belleghem et al. (2017) [75] approved that IL-10 is an anti-inflammatory cytokine that inhibits TH1 cells, NK cells, and macrophage activities [76]. It blocks the expression of pro-inflammatory cytokines, embracing TNF-α, IL-12, IL-1, IFN-γ, and CXC and CC chemokines. The suppression of these cytokines is clearly visible against *S. aureus* and *P. aeruginosa*. On the other hand, Leech et al. (2017) [77] documented that when *S. aureus* is acutely infected, the induction of *IL-10* significantly affects the course of the infection. Too much *IL-10* at one end of the scale may suppress otherwise protective T cell responses, thus simplifying the persistence of the bacteria, and at the other end, too little *IL-10* may tend toward fatal host-mediated pathology via excessive activation of T cells and associated phagocyte-mediated damage.

## 5. Conclusions

As a first report, *Lagenaria siceraria* or *Thymus vulgaris* loaded with chitosan nanoparticles showed tremendous antimicrobial, anti-inflammatory, and antioxidant properties, suggesting their potential for handling inflammatory situations and oxidative stress-related disorders.

## Figures and Tables

**Table 1:** Antimicrobial resistance of *P. aeruginosa* and VRSA strains (n = 10 each) under study.

Antimicrobial Class	AMA	No. of Resistant Strains (%)	MAR Index	*p*-Value
*P. aeruginosa* strains (n = 10)				
Aminoglycosides	CN	7 (70.00)	0.04	0.0736
	AK	5 (50.00)	0.03	1.00
	NET	6 (60.00)	0.35	0.3710
	TOB	7 (70.00)	0.04	0.0736
Carbapenems	IMP	0 (0.00)	0	ND
	MRP	1 (10.00)	0.005	**0.0003**
Cephalosporins	FOX	10 (100.0)	0.06	ND
	CAZ	10 (100.0)	0.06	ND
	FEB	5 (50.00)	0.03	1.00
Quinolones	NA	8 (80.00)	0.05	**0.0072**
	CIP	9 (90.00)	0.05	**0.0003**
	LEV	8 (80.00)	0.05	**0.0072**
Penicillin	PTZ	1 (10.00)	0.005	**0.0003**
Sulphonamide	SXT	10 (100.0)	0.06	ND
Monobactam	ATM	7 (70.00)	0.04	0.0736
Phosphonic	FF	10 (100.0)	0.06	ND
polymyxin	PB	7 (70.00)	0.04	0.0736
VRSA strains (n = 10)				
Aminoglycosides	CN	3 (30.00)	0.02	0.0736
	S	3 (30.00)	0.02	0.0736
Rifamycin	RA	2 (20.00)	0.01	**0.0072**
Cephalosporins	FOX	10 (100.0)	0.06	ND
Penicillin	AX	10 (100.0)	0.06	ND
	AMP	10 (100.0)	0.06	ND
Glycopeptides	VA	8 (80.00)	0.05	**0.0072**
Fluoroquinolones	CIP	3 (30.00)	0.02	0.0736
Tetracyclines	TC	4 (40.00)	0.03	0.3710
	DO	3 (30.00)	0.02	0.0736
Sulphonamides	SXT	7 (70.00)	0.04	0.0736
Carbapenems	IMP	0 (0.00)	0	ND
Macrolide	E	10 (100.0)	0.06	ND
Chloramphenicol	C	10 (100.0	0.06	ND
Phosphonic	FF	10 (100.0)	0.06	ND
Fusidane	FA	9 (90.00)	0.05	**0.0003**

The inhibition zone obtained by each antimicrobial was recorded by mm and interpreted according to CLSI (2021). CN, gentamicin; AK, amikacin; NET, netilmicin; TOB, tobramycin; IMP, imipenem; MRP, meropenem; FOX, cefoxitin; FEB, cefepime; CAZ, ceftazidime; CIP, ciprofloxacin; LEV, levofloxacin; NA, nalidixic acid; PTZ, piperacillin-tazobactam; SXT, trimethoprim + sulfamethoxazole; ATM, aztreonam; FF, fosfomycin; VRSA, vancomycin-resistant *Staphylococcus aureus*; S, streptomycin; RA, rifampin; AX, amoxicillin; AMP, ampicillin; VA, vancomycin; TC, tetracycline; DO, doxycycline; E, erythromycin; C, chloramphenicol; FF, fosfomycin; FA, fusidic acid; MAR, multiple antibiotic resistance; ND, not detected. Data were analyzed by the Fisher exact test. Bold values indicate significant differences at *p* < 0.05.

**Table 2:** Chemical constituents of *Lagenaria siceraria* by gas chromatography–mass spectrometry.

Peak	Compound	Formula	Retention Time (min)	Area under Peak	%
1	Hexadecanoic acid, methyl ester	C_17_H_34_O_2_	17.25	2,340,683.91	4.93
2	9,12-Octadecadienoic acid (Z,Z)-, methyl ester	C_19_H_34_O_2_	19.111	39,671,581.83	83.5
3	9-Octadecenoic acid (Z)-, methyl ester	C_19_H_36_O_2_	19.154	4,782,642.89	10.07
4	Octadecanoic acid, methyl ester	C_19_H_38_O_2_	19.362	715,454.13	1.51

**Table 3:** Chemical constituents of *Thymus vulgaris* by gas chromatography–mass spectrometry.

Peak	Compound	Formula	Retention Time (min)	Area under Peak	%
1	3-Carene	C_10_H_16_	4.43	714,943.46	7.45
2	Benzene, 1-methyl-3-(1-methylethyl)-	C_10_H_14_	5.86	1,292,251.84	13.46
3	D-Limonene	C_10_H_16_	5.917	2,559,940.08	26.67
4	Eucalyptol	C_10_H_18_O	6.038	437,115.8	4.55
5	Gamma-Terpinene	C_10_H_16_	6.375	272,565.1	2.84
6	Bicyclo[2.2.1]heptan-2-one, 1,7,7-trimethyl-, (1S)-	C_10_H_16_O	7.817	594,544.71	6.19
7	Terpinen-4-ol	C_10_H_18_O	8.286	1,028,966.54	10.72
8	Alpha-Terpineol	C_10_H_18_O	8.509	287,248.53	2.99
9	N-Methyl-1-adamantaneacetamide	C_13_H_21_NO	9.242	160,158.78	1.67
10	Isobornyl acetate	C_12_H_20_O_2_	9.785	1,156,405.17	12.05
11	Alpha-Terpinyl acetate	C_12_H_20_O_2_	10.581	933,090.12	9.72
12	Di-n-decylsulfone	C_20_H_42_O_2_S	12.578	161,638.51	1.68

**Table 4:** The antimicrobial activities of *Lagenaria siceraria* and *Thymus vulgaris* essential oils and their nanoparticles on *P. aeruginosa* and VRSA strains (n = 10 each).

Bacterial Strains	Code No.	*Lagenaria siceraria*					*L. siceraria* + Chitosan NPs					*Thymus vulgaris*					*T. vulgaris* + Chitosan NPs				
		Zone Diameters (mm)			Broth Microdilution (µg/mL)		Zone Diameters (mm)			Broth Microdilution (µg/mL)		Zone Diameters (mm)			Broth Microdilution (µg/mL)		Zone Diameters (mm)			Broth Microdilution (µg/mL)	
*P. aeruginosa*		20%	60%	100%	MIC	MBC	20%	60%	100%	MIC	MBC	20%	60%	100%	MIC	MBC	20%	60%	100%	MIC	MBC
	1P	0	2	15	128	256	30	40	50	0.5	1	0	6	14	128	256	20	30	35	4	8
	2P	0	0	10	128	256	28	38	45	0.5	1	0	4	14	128	256	25	30	40	4	8
	3P	0	6	18	64	128	32	40	50	0.5	1	0	5	15	64	64	10	17	25	2	4
	4P	0	4	16	64	128	30	42	50	1	2	0	5	16	64	128	20	35	42	8	16
	5P	0	8	20	128	256	32	44	48	1	2	0	8	18	64	128	20	30	40	4	8
	6P	0	2	12	64	128	24	32	44	0.5	1	0	6	16	32	64	15	22	30	4	8
	7P	0	4	14	64	128	30	35	45	0.25	0.5	0	6	15	128	256	20	24	32	4	8
	8P	0	4	14	128	256	28	34	44	1	2	0	5	12	64	128	24	32	40	4	8
	9P	0	0	6	64	128	25	38	46	0.5	1	0	0	10	64	128	25	30	38	8	16
	10P	0	2	6	128	256	32	40	50	1	2	0	4	16	128	256	25	32	40	8	16
VRSA	1V	0	2	15	128	256	30	40	50	0.25	0.5	0	4	12	32	64	20	32	42	2	4
	2V	0	0	10	128	256	28	38	45	0.25	0.5	0	0	10	64	128	18	30	40	2	4
	3V	0	6	18	128	256	32	40	50	0.125	0.25	0	6	12	128	256	18	30	36	1	2
	4V	0	4	16	32	64	30	42	50	0.25	0.5	0	6	14	16	32	22	32	38	2	4
	5V	0	8	20	64	128	32	44	48	0.25	0.5	0	8	20	64	128	25	35	45	2	4
	6V	0	2	12	128	256	24	32	44	0.5	1	0	6	16	128	256	20	28	32	2	4
	7V	0	4	14	64	128	30	35	45	0.5	1	0	5	14	64	128	18	30	38	4	8
	8V	0	4	14	64	128	28	34	44	0.125	0.25	0	4	12	64	128	22	30	40	2	4
	9V	0	0	6	128	256	25	38	46	0.25	0.5	0	5	10	64	128	14	26	32	2	4
	10V	0	2	6	64	128	32	40	50	0.5	1	0	6	12	64	128	16	28	38	4	8

VRSA, vancomycin resistant *Staphylococcus aureus*; MIC, minimum inhibitory concentration, MBC, minimum bactericidal concentration; NPs, nanoparticles.

**Table 5:** Changes in serum biochemical and redox status of rats infected by *P. aeruginosa* and VRSA strains within each group.

Groups	ALT (IU/dL)	AST (IU/dL)	Alb (g/dL)	Creatinine (mg/dL)	Urea (mg/dL)	SOD (U/mL)	GPX (MU/mL)	MDA (mmol/mL)
G1	42.97 ± 1.36 ^b^	121.33 ± 2.96 ^b^	3.52 ± 0.03 ^a^	0.57 ± 0.01 ^b^	24.13 ± 5.52 ^a,b^	126.33 ± 0.88 ^a^	19.26 ± 0.57 ^a^	3.27 ± 0.23 ^f^
G2	58.60 ± 0.56 ^a^	155.33 ± 2.60 ^a^	3.07 ± 0.04 ^c^	0.66 ± 0.02 ^a^	25.80 ± 6.31 ^a,b^	62.67 ± 1.45 ^e^	2.62 ± 0.33 ^e^	14.77 ± 0.30 ^a^
G3	58.97 ± 1.03 ^a^	146.67 ± 2.03 ^a^	3.27 ± 0.04 ^b^	0.64 ± 0.03 ^a^	14.39 ± 1.86 ^b^	68.00 ± 2.31 ^d^	5.63 ± 0.75 ^d^	12.03 ± 0.43 ^b^
G4	40.43 ± 2.78 ^b^	132.33 ± 5.36 ^b^	3.35 ± 0.03 ^b^	0.68 ± 0.01 ^a^	19.18 ± 2.21 ^a,b^	97.67 ± 1.20 ^c^	9.24 ± 0.61 ^c^	10.87 ± 0.30 ^c^
G5	41.70 ± 3.65 ^b^	127.67 ± 4.06 ^b^	3.57 ± 0.04 ^a^	0.52 ± 0.02 ^b^	15.25 ± 1.93 ^b^	103.67 ± 0.84 ^b^	12.80 ± 0.64 ^b^	7.13 ± 0.48 ^e^
G6	42.23 ± 1.63 ^b^	130.67 ± 3.28 ^b^	3.32 ± 0.06 ^b^	0.66 ± 1.01 ^a^	36.03 ± 4.72 ^a^	97.33 ± 1.20 ^c^	9.42 ± 0.49 ^c^	9.63 ± 0.23 ^d^
G7	60.40 ± 1.72 ^A^	148.67 ± 3.76 ^A^	3.31 ± 0.05 ^B,C^	0.65 ± 0.01 ^A^	18.99 ± 0.73	69.33 ± 2.33 ^F^	4.77 ± 0.24 ^E^	15.87 ± 0.38 ^A^
G8	54.53 ± 0.88 ^A^	148.67 ± 4.10 ^A^	3.48 ± 0.06 ^A,B^	0.65 ± 0.01 ^A^	24.43 ± 2.69	78.00 ± 1.15 ^E^	7.97 ± 0.41 ^D^	13.13 ± 0.48 ^B^
G9	56.73 ± 2.70 ^A^	143.33 ± 5.24 ^A^	3.23 ± 0.10 ^C^	0.61 ± 0.03 ^A,B^	31.80 ± 7.68	93.67 ± 0.88 ^D^	10.40 ± 0.29 ^C^	11.20 ± 0.46 ^C^
G10	40.00 ± 2.08 ^B^	116.00 ± 3.79 ^B^	3.48 ± 0.02 ^A,B^	0.63 ± 0.01 ^A,B^	24.78 ± 1.56	108.00 ± 2.08 ^B^	12.83 ± 0.91 ^B^	7.37 ± 0.61 ^E^
G11	41.30 ± 1.42 ^B^	123.67 ± 2.73 ^B^	3.35 ± 0.03 ^A,B,C^	0.63 ± 0.04 ^A,B^	20.35 ± 2.32	98.33 ± 0.81 ^C^	12.10 ± 0.97 ^B,C^	9.20 ± 0.35 ^D^
*p*-value	0.0005	<0.0001	0.0189	0.0004	0.0500	<0.0001	<0.0001	<0.0001

G1, negative control (non-infected non-treated group); G2, *P. aeruginosa* infected group; G3, *P. aeruginosa* infected then treated with imipenem; G4, *P. aeruginosa* infected then treated with chitosan; G5, *P. aeruginosa* infected then treated with *Lagenaria siceraria* loaded chitosan nanoparticles; G6, *P. aeruginosa* infected then treated with *Thymus vulgaris* loaded chitosan nanoparticles; G7, VRSA infected group; G8, VRSA infected then treated with imipenem; G9, VRSA infected then treated with chitosan; G10, VRSA infected then treated with *Lagenaria siceraria* loaded chitosan nanoparticles; G11, VRSA infected then treated with *Thymus vulgaris* loaded chitosan nanoparticles; VRSA, vancomycin-resistant *S. aureus*; ALT, alanine transaminase; AST, aspartate aminotransferase; Alb, albumin; SOD, superoxide dismutase; GPX, glutathione peroxidase; MDA, serum malondialdehyde. Data were analyzed by a One-way ANOVA analysis and Tukey’s test. ^a,b,c,d,e^ Mean values (for negative control (G1) and *P. aeruginosa* groups (G2–G6) followed by different superscript small letters in the same column are significantly different (*p* < 0.05). ^A,B,C,D,E^ Mean values (for *S. aureus* groups, G7–G11) followed by different superscript capital letters in the same column are significantly different (*p* < 0.05).

**Table 6:** Changes in blood hematology of rats infected by *P. aeruginosa* and VRSA within each group.

Groups	Hb (g/dL)	RBCs (10^6^/mm^3^)	WBCs (10^9^/L)	NEUT (%)	LYM (%)
G1	11.77 ± 0.02 ^a^	6.87 ± 0.02 ^a^	9.67 ± 0.09 ^e^	17.33 ± 1.20 ^e^	83.33 ± 1.20 ^a^
G2	9.35 ± 0.09 ^c^	5.37 ± 0.08 ^c^	23.17 ± 0.59 ^a^	39.33 ± 1.19 ^a^	62.33 ± 1.20 ^d^
G3	9.58 ± 0.04 ^c^	5.84 ± 0.02 ^b^	18.23 ± 0.19 ^b^	33.00 ± 1.15 ^b^	72.67 ± 2.33 ^bc^
G4	10.18 ± 0.12 ^b^	5.89 ± 0.10 ^b^	15.37 ± 0.29 ^c^	28.33 ± 1.21 ^c^	72.67 ± 0.88 ^bc^
G5	11.60 ± 0.14 ^a^	6.68 ± 0.08 ^a^	12.60 ± 0.74 ^d^	22.33 ± 0.87 ^d^	76.33 ± 1.45 ^b^
G6	10.41 ± 0.41 ^b^	6.08 ± 0.11 ^b^	14.87 ± 0.18 ^c^	29.00 ± 0.12 ^c^	69.33 ± 0.87 ^c^
G7	9.41 ± 0.06 ^C^	5.41 ± 0.02 ^E^	18.37 ± 0.50 ^A^	33.00 ± 0.58 ^A^	68.33 ± 0.84 ^D^
G8	10.67 ± 0.22 ^B^	6.26 ± 0.06 ^C^	13.80 ± 0.38 ^BC^	25.67 ± 0.67 ^B^	73.33 ± 0.82 ^C^
G9	9.63 ± 0.11 ^C^	5.61 ± 0.06 ^D^	14.83 ± 0.35 ^B^	28.33 ± 0.88 ^B^	71.00 ± 0.58 ^C^
G10	11.64 ± 0.09 ^A^	6.47 ± 0.03 ^B^	9.87 ± 0.34 ^D^	21.67 ± 0.74 ^C^	76.67 ± 0.80 ^B^
G11	10.57 ± 0.26 ^B^	6.14 ± 0.12 ^C^	12.87 ± 0.35 ^C^	26.00 ± 0.58 ^B^	73.00 ± 0.57 ^C^
*p*-value	<0.0001	<0.0001	<0.0001	<0.0001	<0.0001

G1, negative control (non-infected non-treated group); G2, *P. aeruginosa* infected group; G3, *P. aeruginosa* infected then treated with imipenem; G4, *P. aeruginosa* infected then treated with chitosan; G5, *P. aeruginosa* infected then treated with *Lagenaria siceraria* loaded chitosan nanoparticles; G6, *P. aeruginosa* infected then treated with *Thymus vulgaris* loaded chitosan nanoparticles; G7, VRSA infected group; G8, VRSA infected then treated with imipenem; G9, VRSA infected then treated with chitosan; G10, VRSA infected then treated with *Lagenaria siceraria* loaded chitosan nanoparticles; G11, VRSA infected then treated with *Thymus vulgaris* loaded chitosan nanoparticles; VRSA, vancomycin-resistant *S. aureus*; Hb, hemoglobulin; RBCs, red blood cells; WBCs, white blood cells; NEUT, Neutrophils; LYM, Lymphocytes. Data were analyzed by a One-way ANOVA analysis and Tukey’s test. ^a,b,c,d,e^ Mean values (for negative control (G1) and *P. aeruginosa* groups (G2–G6) followed by different superscript smaller letters in the same column are significantly different (*p* < 0.05). ^A,B,C,D,E^ Mean values (for *S. aureus* groups, G7–G11) followed by different superscript capital letters in the same column are significantly different (*p* < 0.05).

## Data Availability

The data presented in this study are available upon request from the corresponding author.

## Supplementary Materials

The following supporting information can be downloaded at https://www.mdpi.com/article/10.3390/antiox13040428/s1. Figure S1: Postmortem changes in the positive control groups showed abscess formation on kidney (A), spleen (A), intestine (B), and liver (C) compared with rats treated by chitosan nanoparticles loaded with either *Lagenaria siceraria* oil or *Thymus vulgaris*, which showed normal internal organs (D). Figure S2. Photomicrograph from livers, kidneys and spleens of rats infected with *P. aeruginosa* then treated with imipenem (A) and chitosan nanoparticles (B). A: It shows mild portal round cells (lymphocytes and plasma cells) aggregation (blue arrows) and focal hepatocellular degeneration, mainly hydropic degeneration (black arrows). Kidney sections show mild renal vascular dilatation with blood engorgement (green star) interstitial, periglomerular lymphocytic aggregations (white stars), focal glomerular shrinkage and sclerosis (black arrow), renal tubular epithelial degeneration (blue arrow) and necrotizing pyelitis (yellow arrow). Spleen shows apparently normal white (black, orange circles, blue arrows, green, black stars) and red pulp (red stars). H&E ×100, 200, 400. B: It shows mild portal round cells (lymphocytes and plasma cells) aggregation (blue arrow) and scattered apoptotic cells (black arrow). Kidney sections reveal mild perivascular edema associated with lympho-plasmacytic infiltration and focal glomerular shrinkage with periglomerular lymphocytic aggregations (white stars). Spleen shows normal features of the white (black, orange, blue circles, green and black stars) and red pulp (red star). H&E ×100, 200, 400. Table S1. Oligonucleotide primers used in the study.
