# Supplementary material for: Chitosan-Loaded Lagenaria siceraria and Thymus vulgaris Potentiate Antibacterial, Antioxidant, and Immunomodulatory Activities against Extensive Drug-Resistant Pseudomonas aeruginosa and Vancomycin-Resistant Staphylococcus aureus: In Vitro and In Vivo Approaches
Source: Antioxidants (Basel). 2024 Mar 30;13(4):428. doi: 10.3390/antiox13040428 (PMC11047512; doi:10.3390/antiox13040428)
Supplement: Supplementary file 1 [file antioxidants-13-00428-s001.zip › Table S1.pdf]

Supplementary Table 1. Oligonucleotide primers used in the study.

Target gene	Primers sequences 5 → 3`	Specificity	Annealing temperature (° C)	References
oprL	F: ATGGAAATGCTGAAATTCGGC<br>R: CTTCTTCAGCTCGACGCGACG	*P. aeruginosa* species-specific primer	**57**	**[18]**
nuc	F: GCGATTGATGGTGATACGGTI<br>R: AGCCAAGCCTTGACGAACTAAAGC	*S. aureus* species-specific primer	**55**	**[19]**
IL10	F: GCGGCTGAGGCGCTGTCAT<br>R: CGCCTTGTAGACACCTTGGTCTTGG	Cytokine gene	**60**	**[44]**
IFNG	F: AACGCTACACACTGCATCTTGG<br>R: GACTTCAAAGAGTCTGAGG	Cytokine gene	**55**	**[45]**
ß-actin	F: TCCTCCTGAGCGCAAGTACTCT<br>R: GCTCAGTAACAGTCCGCCTAGAA	A housekeeping gene	**60**	**[43]**

F, forward; R, reverse; Bp base pair.
