# Supplementary figures and images for: Chitosan-Loaded Lagenaria siceraria and Thymus vulgaris Potentiate Antibacterial, Antioxidant, and Immunomodulatory Activities against Extensive Drug-Resistant Pseudomonas aeruginosa and Vancomycin-Resistant Staphylococcus aureus: In Vitro and In Vivo Approaches
Source: Antioxidants (Basel). 2024 Mar 30;13(4):428. doi: 10.3390/antiox13040428 (PMC11047512; doi:10.3390/antiox13040428)

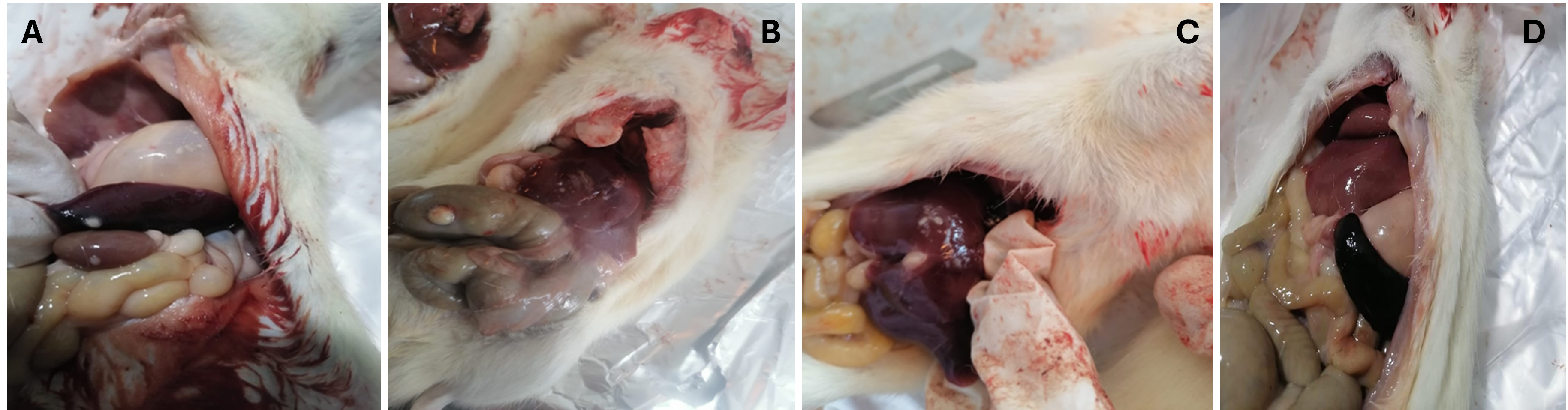

Supplement: Supplementary file 1 [file antioxidants-13-00428-s001.zip › Supplementary Figure 1.tif]

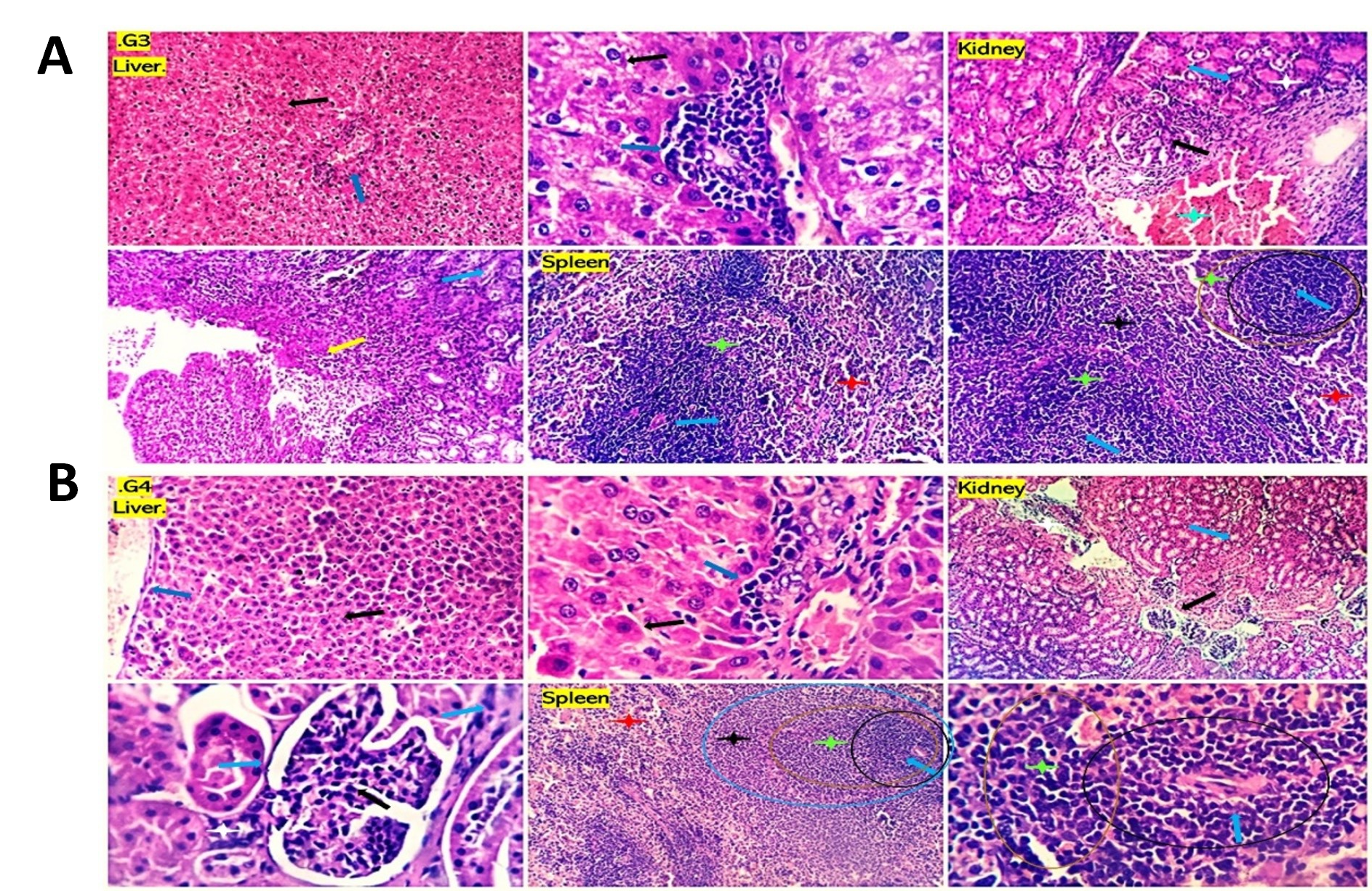

Supplement: Supplementary file 1 [file antioxidants-13-00428-s001.zip › Supplementary Figure 2.tif]
